# Lysosome restoration to activate podocyte autophagy: a new therapeutic strategy for diabetic kidney disease

**DOI:** 10.1038/s41419-019-2002-6

**Published:** 2019-10-24

**Authors:** Wei Jing Liu, Yu Gan, Wei Fang Huang, Hong-luan Wu, Xue-qin Zhang, Hui Juan Zheng, Hua-feng Liu

**Affiliations:** 1 Institute of Nephrology, and Zhanjiang Key Laboratory of Prevention and Management of Chronic Kidney Disease, Guangdong Medical University, Zhanjiang, 524001 Guangdong China; 2 Key Laboratory of Chinese Internal Medicine of Ministry of Education and Beijing, Dongzhimen Hospital Affiliated to Beijing University of Chinese Medicine, Shipping warehouse No. 5, 100700 Beijing, China

**Keywords:** Macroautophagy, Lysosomes

## Abstract

Autophagy, the intracellular lysosomal degradation process plays a pivotal role in podocyte homeostasis in diabetic kidney disease (DKD). Lysosomal function, autophagic activity, and their actions were investigated in vitro and in vivo. We found that LC3-II- and p62-positive vacuoles accumulated in podocytes of patients with DKD. Moreover, we found that advanced glycation end products (AGEs) could increase the protein expression of LC3-II and p62 in a dose- and time-dependent manner in cultured podocytes. However, the mRNA expression of LC3B, Beclin-1 or ATG7, as well as the protein level of Beclin-1 or ATG7 did not change significantly in the AGE-treated cells compared with that in control groups, suggesting that AGEs did not induce autophagy. In addition, AGEs led to an increase in the number of autophagosomes but not autolysosomes, accompanied with a failure in lysosomal turnover of LC3-II or p62, indicating that the degradation of autophagic vacuoles was blocked. Furthermore, we observed a dramatic decrease in the enzymatic activities, and the degradation of DQ-ovalbumin was significantly suppressed after podocytes were treated with AGEs. Plasma-irregular lysosomal-associated membrane protein 1 granules accompanied with the diffusion of cathepsin D expression and acridine orange redistribution were observed in AGE-treated podocytes, indicating that the lysosomal membrane permeability was triggered. Interestingly, we also found that AGEs-induced autophagic inhibition and podocyte injury were mimicked by the specific lysosomotropic agent, l-leucyl-l-leucine methyl ester. The exacerbated apoptosis and Rac-1-dependent actin-cytoskeletal disorganization were alleviated by an improvement in the lysosomal-dependent autophagic pathway by resveratrol plus vitamin E treatment in AGE-treated podocytes. However, the rescued effects were reversed by the addition of leupeptin, a lysosomal inhibitor. It suggests that restoring lysosomal function to activate autophagy may contribute to the development of new therapeutic strategies for DKD.

## Introduction

Diabetic kidney disease (DKD), a major devastating complication of diabetes, is becoming a serious health problem worldwide owing to its rapidly increasing prevalence, poor prognosis, and heavy economic burden^1^. DKD implicates the alterations of almost all of the renal intrinsic cells, resulting in many pathological processes such as the hypertrophy of glomerularor tubular cells, glomerulosclerosis, and tubulointerstitial fibrosis^2^. Podocytes, attached to the outer layer of the glomerular filtration barrier (GFB), are pivotal in maintaining the integrity of GFB^3^. They are terminally differentiated cells with no ability to proliferate in vivo; therefore, the injury and loss of podocytes contribute much to proteinuria and glomerulosclerosis during the progression of DKD^4–6^. Various metabolites can result in damage to podocytes, such as advanced glycation end products (AGEs), which could interact with the receptor of AGE (RAGE) to initiate downstream signaling pathways, such as inflammation, interaction with rennin–angiotensin system (RAS), and oxidative stress, resulting in the pathogenesis of DKD^7,8^. Although some potential targets for renoprotective therapies have been studied and tested in experimental and clinical models according to well-known pathways, the efficacy has not been satisfactory^9–11^.

Autophagy is a conserved catabolic process that degrades damaged proteins and organelles to maintain intracellular homeostasis^12^. It is well-known that podocyte homeostasis is maintained by a high level of basal autophagy^13^. It is suggested that the activation of autophagy allows for an adaptive response in podocytes, and may be a potential new therapeutic target for DKD^14^. Previous study has demonstrated that autophagy-deficient mice fail to induce lysosomal biogenesis, causing an accumulation of AGEs in renal intrinsic cell and the cellular injury^15^. Autophagy also plays a pivotal role in keeping lysosome homostasis and the blockage of autophagy induction results in an accumulation of huge damaged lysosomes (lysophagy), followed by podocyte apoptosis in DKD^16^. It reveals the important role of autophagy induction in maintaining lysosomal function and protecting podocytes from injury in diabetes condition. However, the autophagic process is a very complicated pathway, including autophagic induction, fusion between autophagic vacuoles and lysosomes, and lysosomal degradation of autophagic vacuoles^17^. If any node is damaged or blocked, autophagy will be inactivated. Lysosomes, as the major degradative compartment, are located at the terminal process of the autophagic pathway and play a vital role in autophagic degradation of macromolecules and organelles^18^. Several studies have shown that diabetes could cause a decrease in enzyme activity as well as a reduction in the degradation of albumin in lysosomes^19,20^. Lysosmal dysfuction might be triggered by some etiological factors in DKD, which results in blockage of the downstream pathway of the autophagic process, and thereby leading to podocyte lesion. It is important to illustrate the exact start point and key node of defective autophagy-lysosome pathway, when exploring a specific therapeutic strategy for DKD

## Results

### Autophagic vacuoles accumulated in the podocytes from DKD patients and after exposure to AGEs

**Fig. 1:**
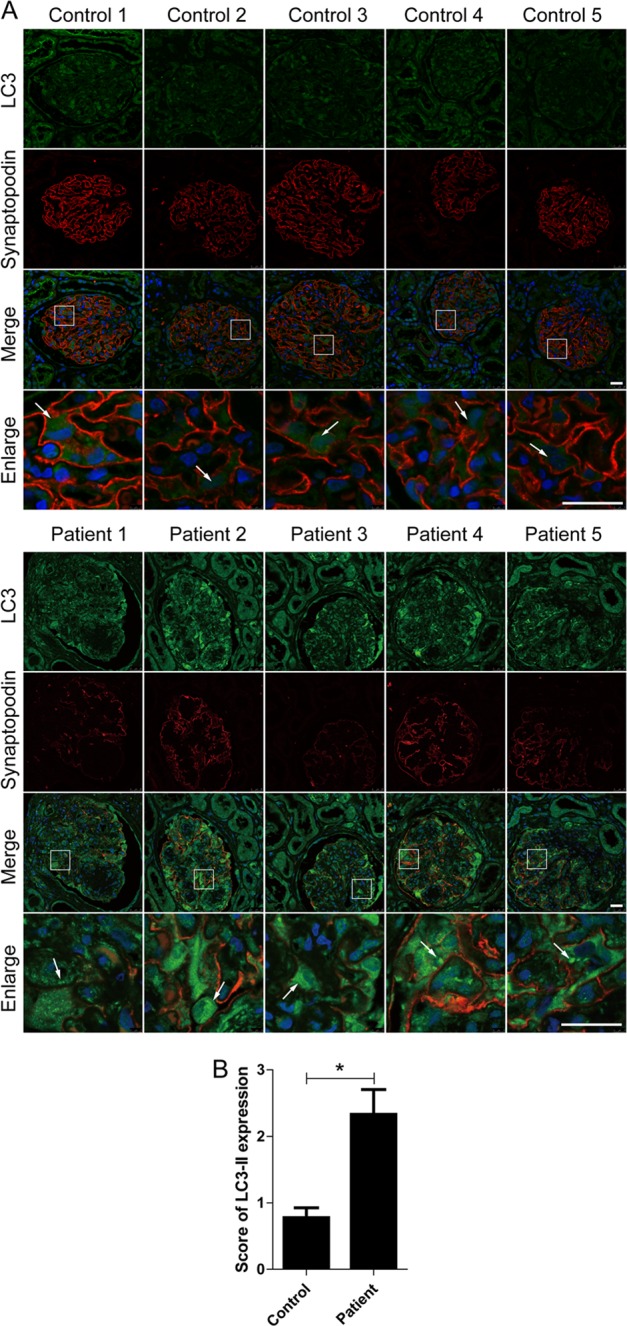
An accumulation of LC3-II in podocytes in patients with DKD. **a** Representative images of immunofluorescence assays of LC3 (green) in podocytes on kidney biopsy specimens from DKD patients (patients 1–5) and controls (controls 1–5). The podocytes in glomeruli were identified by synaptopodin (red), a podocyte-specific protein. The nuclei were stained with DAPI (blue). White arrows indicate the expression of LC3-positive puncta in podocytes. Scale bar, 25 μm. **b** The score of LC3-II expression in podocytes of DKD patients and controls. The expression of LC3-II was increased in podocytes in patients with DKD

**Fig. 2:**
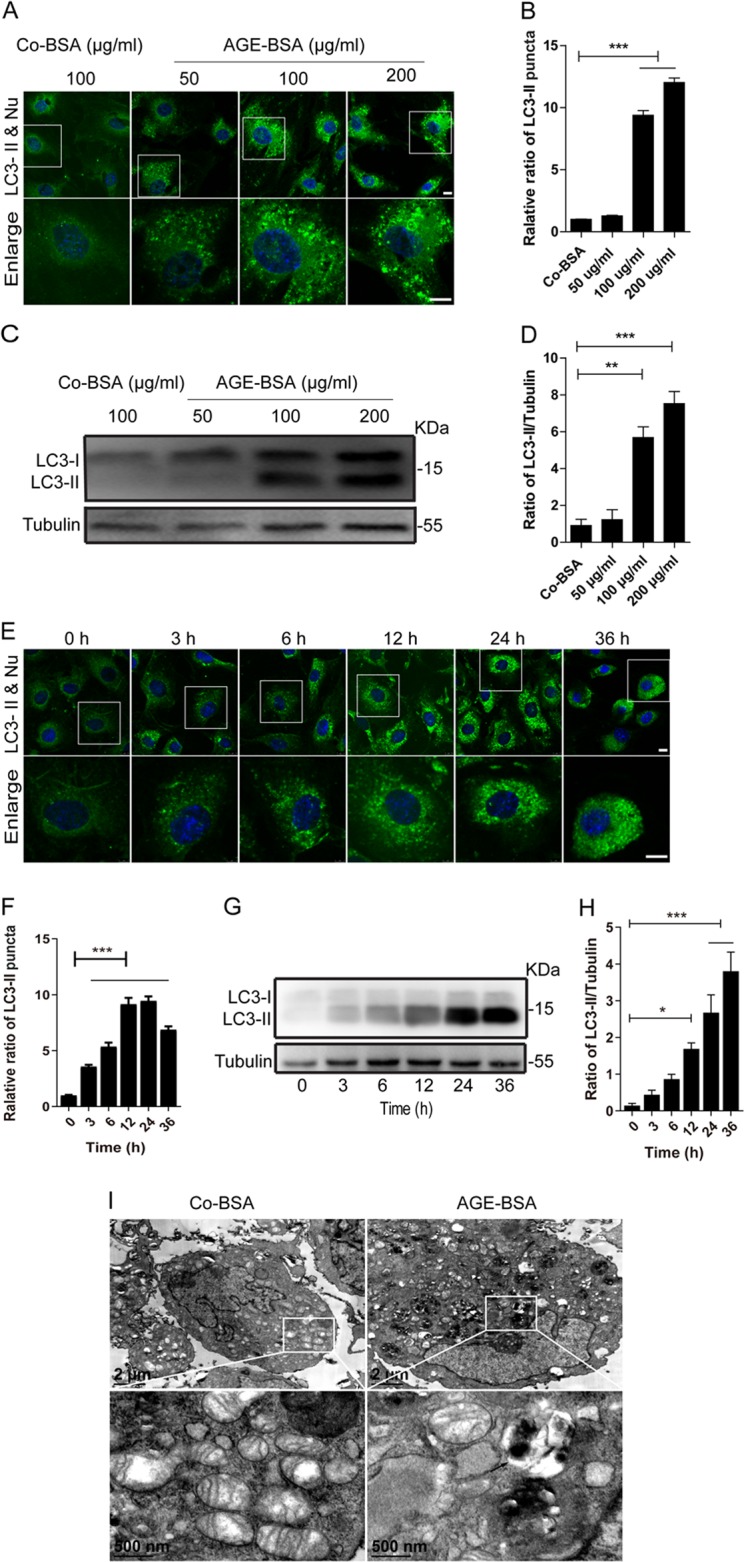
Quantitative changes of autophagic vacuoles after exposure of podocytes to AGE-BSA at different concentrations for different times. **a**, **e** Immunofluorescence staining of LC3 in podocytes after exposure to AGE-BSA at different concentrations and times. Scale bar, 10 μm. **c**, **g** Western blot analysis of LC3. **b**, **d**, **f**, **h** Densitometry was performed for the quantification, and the ratio of LC3-II to tubulin was expressed as the fold-change compared with the level in the control. **i** Ultrastructural images of autophagic vacuoles and mitochondria after exposure of podocytes to AGE-BSA for 12 h. Arrow indicates the autophagic vacuole. AGE-BSA induced the accumulation of autophagic vacuoles in a concentration- and time-dependent manner. *p < 0.05; **p < 0.01; ***p < 0.001

In this study, we first evaluated the expression of microtubule-associated protein 1 light chain 3B (LC3), a key marker of autophagy vacuoles, in podocytes of renal tissues by immunofluorescence. Synaptopodin, the podocyte-specific protein, was marked for basement membranes and podocytes^21^. As shown in Fig. 1a, b, there were more LC3-II-positive puncta in podocytes of DKD patients compared with that in the controls. To simulate DKD in vitro, we incubated podocytes with different concentrations of AGE-BSA (advanced glycation end products-bovine serum albumin) (50, 100, and 200 μg/ml) for 12 h or treated with 100 μg/ml AGE-BSA for different times (3, 6, 12, 24, and 36 h). By immunofluorescence staining, exposure of podocytes to AGE-BSA induced accumulation of LC3-II puncta in a concentration- and time-dependent manner (Fig. 2a, b, e, f). Similar results were obtained by western blotting (Fig. 2c, d, 2g, h). Transmission electron microscopy (TEM) analysis also revealed that autophagic vacuoles, containing partially degraded cytoplasmic material, accumulated in podocytes after exposure to AGE-BSA, which were not detectable in controls (Fig. 2i). In addition, mitochondria ridges became irregular, broken, and disappeared in AGE-BSA-treated podocytes.

### AGEs inhibited autophagic activity in podocytes

**Fig. 3:**
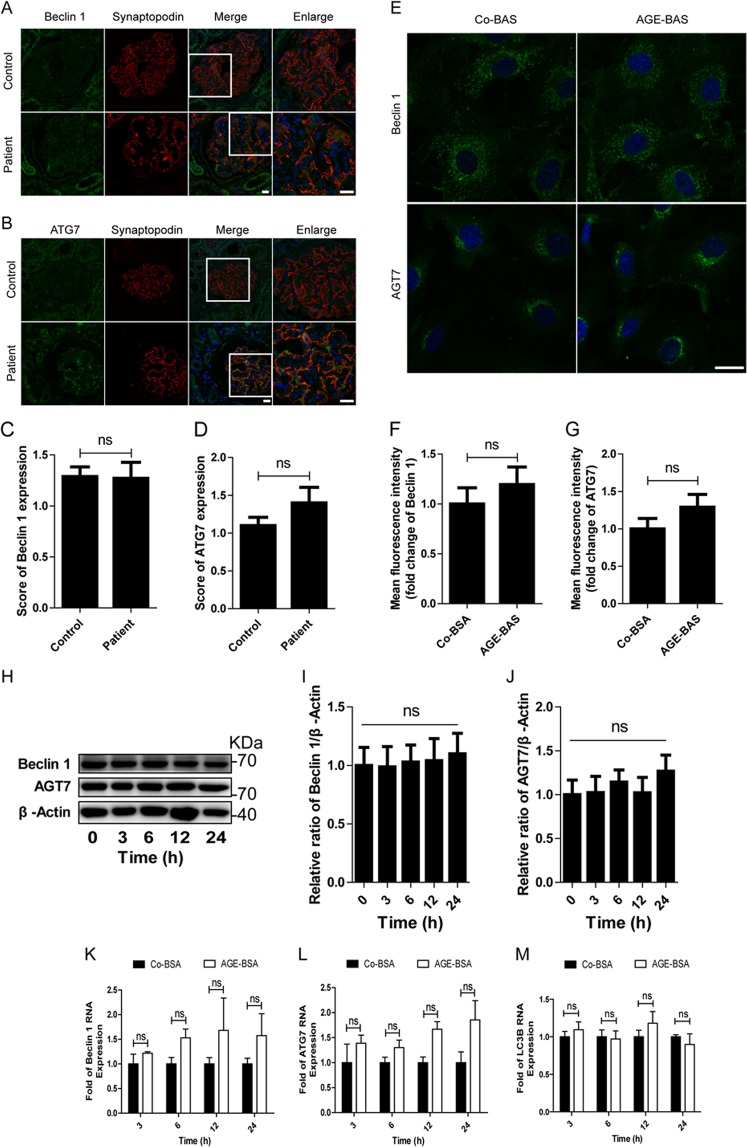
Quantitative changes of protein or mRNA level of Beclin-1 and ATG7 in podocytes from DKD patients and after exposure to AGE-BSA. **a**, **b** Representative images of immunofluorescence assays of Beclin 1 or ATG7 (green) in podocytes on kidney biopsy specimens from DKD patients and controls. The podocytes in glomeruli were identified by synaptopodin (red). The nuclei were stained with DAPI (blue). **c**, **d** The score of Beclin 1 or ATG7 expression in podocytes of DKD patients and controls. **e** Immunofluorescence staining of Beclin 1 or ATG7 after exposure of podocytes to AGE-BSA at 100 μg/ml for 12 h. **f**, **g** Mean fluorescence intensity of Beclin 1 or ATG7 was measured for both groups. **h** Western blot analysis of Beclin 1 or ATG7. **i**, **j** Densitometry was performed for the quantification, and the ratio of Beclin 1 or ATG7 to β-actin was expressed as the fold-change to 0 h. **k**, **l**, **m** The relative mRNA expressions of Beclin-1 (**k**), ATG7 (**l**), and LC3B (**m**) after exposure to AGE-BSA for 3, 6, 12, and 24 h. There was no significant difference observed among all the groups. Scale bar, 20 μm; ns, no significance

Since an accumulation of LC3-II may be caused by increased formation or failure of degradation, we then examined the autophagy inducer Beclin 1 and ATG7. In vivo study, we found that the expression of either Beclin 1 or ATG7 was not changed in podocytes of DKD patients compared with controls, as shown in Fig. 3a–d. In vitro study, the protein levels were not enhanced after exposure of podocytes to AGE-BSA for 12 h (Fig. 3e–g), as quantified by immunofluorescence analysis. Similar results were obtained by western blotting (Fig. 3h–j). In addition, the mRNA levels of Beclin-1, ATG7, and LC3B were not significantly increased at various time points after treatment with 100 μg/ml AGE-BSA (Fig. 3k–m), suggesting that the accumulation of autophagic vacuoles was not due to autophagic induction.

**Fig. 4:**
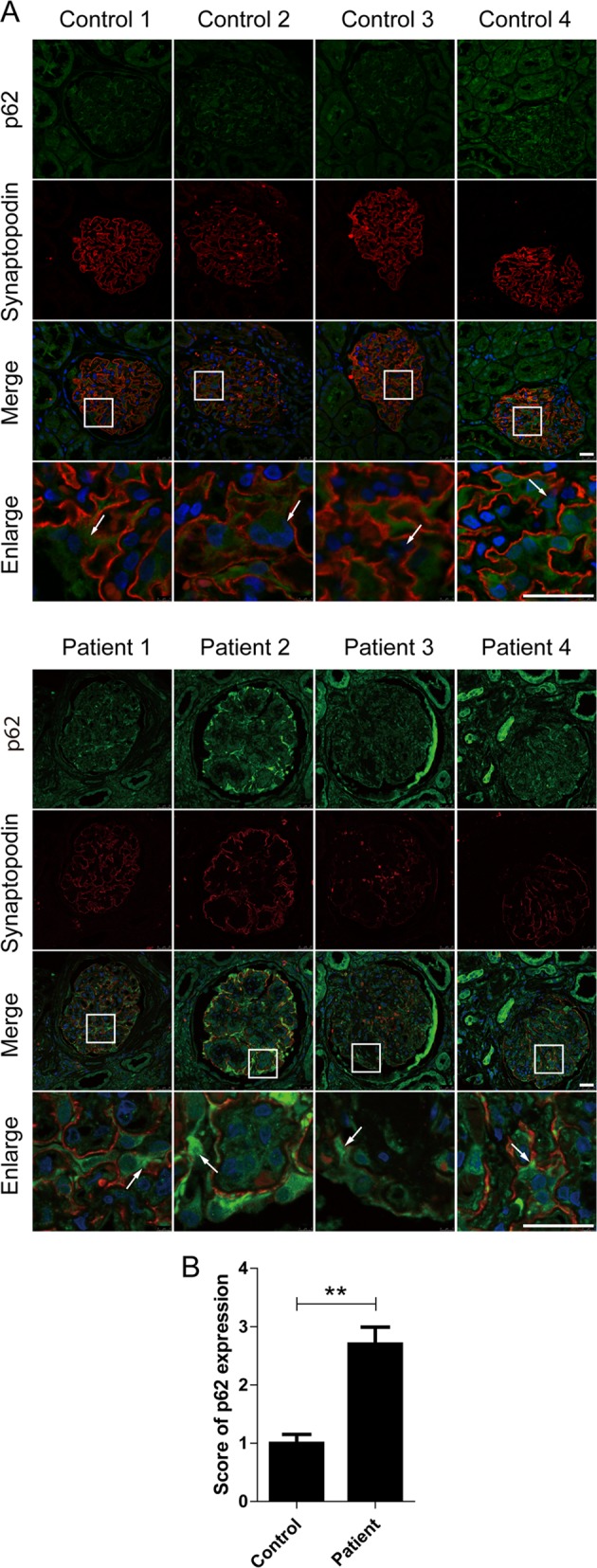
An accumulation of p62 in podocytes in patients with DKD. **a** Representative images of immunofluorescence assays of p62 (green) and synaptopodin (red) proteins in kidney biopsy specimens from a patient with DKD (patient 1–4) and controls (control 1–4). The nuclei were stained with DAPI (blue). White arrows indicate the expression of p62 in podocytes. Scale bar, 25 μm. **b** The score of p62 expression in podocytes of DKD patients and controls. Compared to controls, the expression of p62 was increased in podocytes in patients with DKD

**Fig. 5:**
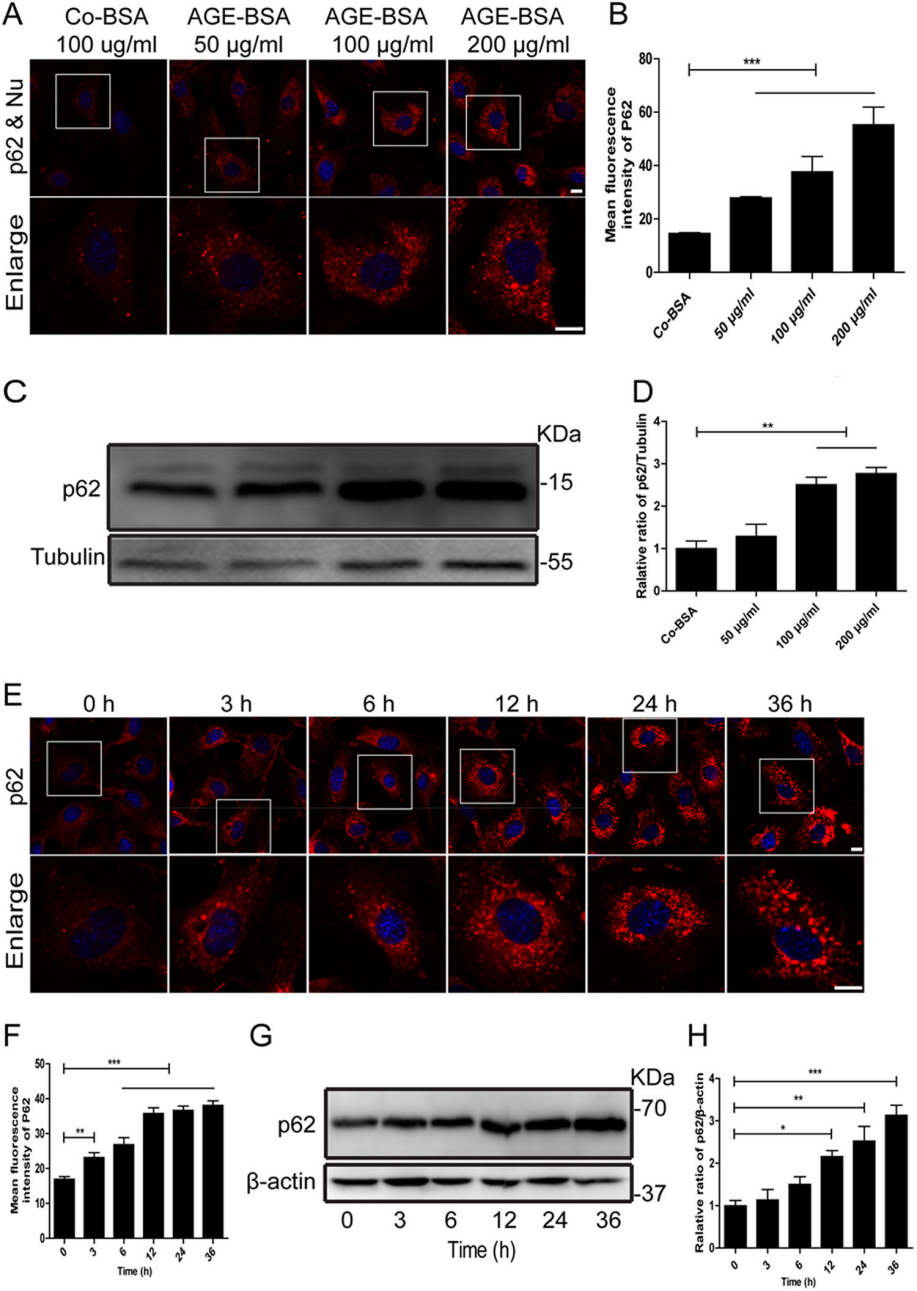
Quantitative changes in autophagy substrate after exposure of podocytes to AGE-BSA at different concentrations for different times. **a**, **e** Immunofluorescence staining of p62 in podocytes after exposure to AGE-BSA at different concentrations and times. **b**, **f** Mean fluorescence intensity of p62 was measured for different groups. **c**, **g** Western blot analysis of p62. **d**, **h** Densitometry was performed for the quantification, and the ratio of p62 to tubulin was expressed as the fold-change compared with the level in the control. The expression of p62 was enhanced in a dose- and time-dependent manner after exposure to AGE-BSA. Scale bar, 10 μm. *p < 0.05; **p < 0.01; ***p < 0.001

Furthermore, p62 has been reported to act as an autophagic receptor and substrate, which binds with ubiquitylated organelles or proteins to autophagosomes for degradation^22,23^. To determine whether the accumulation of autophagy vacuoles correlated with impaired autophagic degradation, we then evaluated the expression of p62 in podocytes both in vivo and in vitro. As shown in Fig. 4a, b, there were more aggregates of green fluorescent spots observed in podocytes in DKD renal tissues, compared with those from the control renal tissues. In vitro, the expression of p62 obviously enhanced after exposure to AGE-BSA with 50, 100, 200 μg/ml and for 3, 6, 12, 24, and 36 h in a dose- and time-dependent manner (Fig. 5a, b, e, f). Consistent results were observed by western blotting (Fig. 5c, d, g, h). These results indicated that AGE-BSA could cause the accumulation of autophagic substrates in podocytes in DKD.

**Fig. 6:**
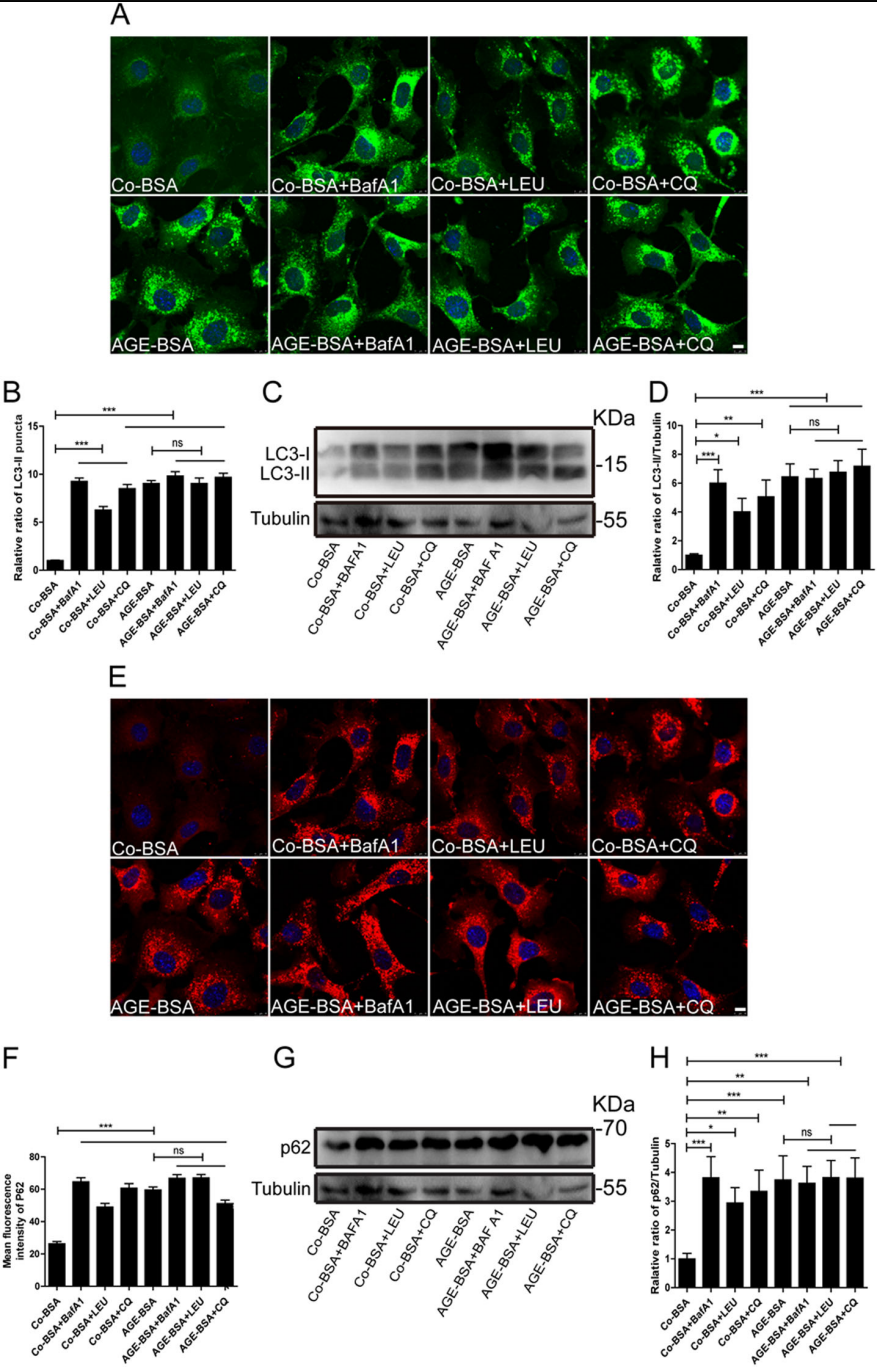
LC3 or p62 turnover assay in vitro. **a**, **b** Immunofluorescence analysis of LC3 and the differences in LC3-II puncta between samples with and without bafilomycin A1 (Baf A1), leupeptin (LEU), and chloroquine (CQ) were compared after exposure to AGE-BSA or Co-BSA. LC3-II turnover (comparing lanes 5, 6, 7, and 8 with 4) was not observed after exposure to AGE-BSA. **c**, **d** Western blot analysis of LC3 and the difference in the LC3-II levels between samples with and without Baf A1, LEU, and CQ was compared after exposure to AGE-BSA or Co-BSA. **e**, **f** Immunofluorescence analysis of p62 and the difference in the mean fluorescence intensity between samples with and without Baf A1, LEU, and CQ was compared after exposure to AGE-BSA or Co-BSA. P62 turnover (comparing lanes 5, 6, 7, and 8 with 4) was not observed after exposure to AGE-BSA. **g**, **h** Western blot analysis of p62 and the difference in the p62 levels between samples with and without Baf A1, LEU, and CQ was compared after exposure to AGE-BSA or Co-BSA. Scale bar, 10 μm. *p < 0.05, **p < 0.01, and ***p < 0.001

LC3-II and p62 turnover was then tested by measuring the amount of LC3-II and p62 delivered to the lysosomes in the presence and absence of lysosomal inhibitors^17^. Bafilomycin A1, leupeptin, and chloroquine are typical lysosomal inhibitors^24^. Both immunofluorescence staining and western blotting showed that LC3-II and p62 expressions did not increase in AGE-BSA plus inhibitors-treated cells compared with those in cells treated with AGE-BSA alone. These data indicate the impairment of autophagy and failure to degrade the autophagosome upon the exposure of podocytes to AGE-BSA (Fig. 6).

**Fig. 7:**
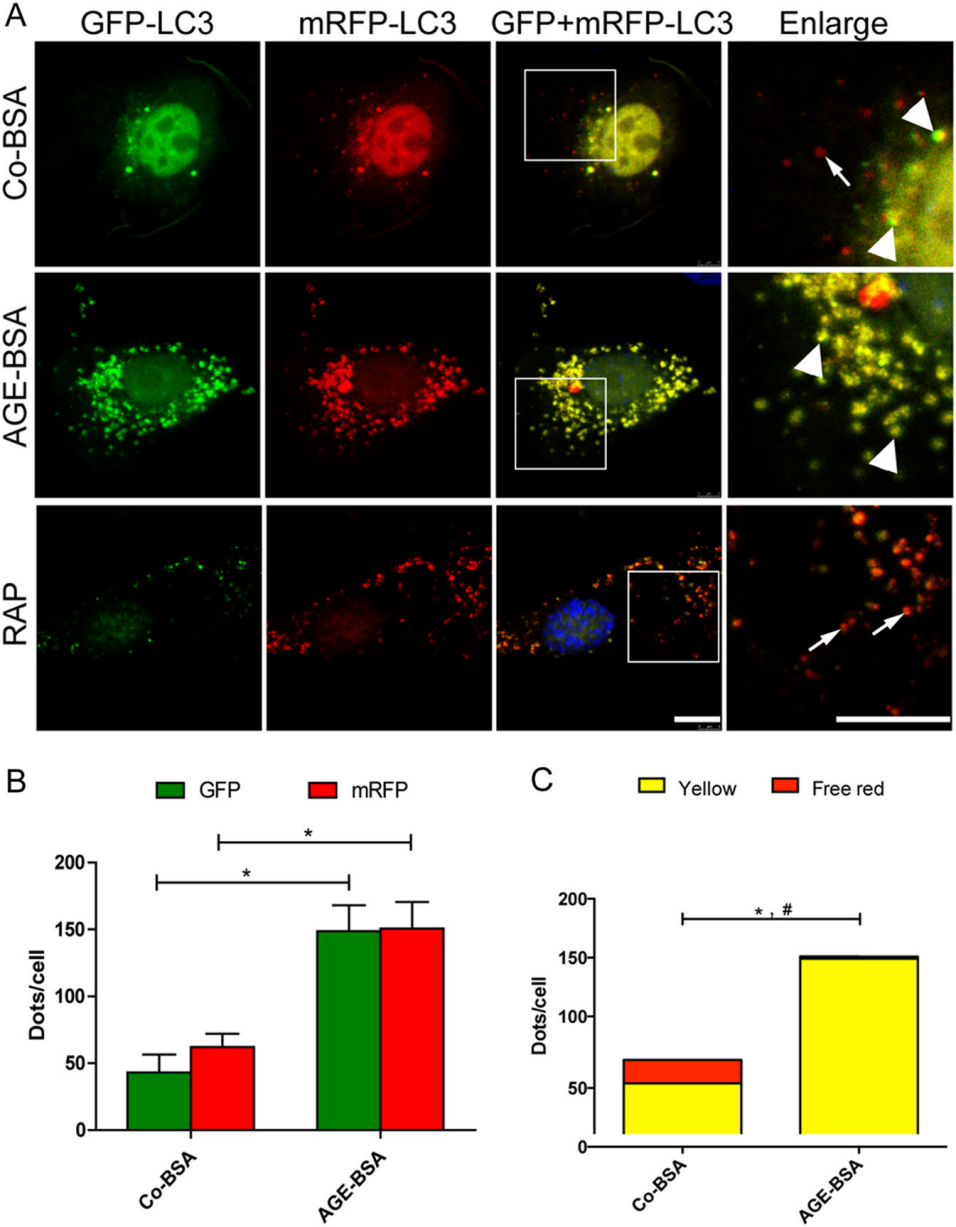
Quantitative changes in autophagosomes and autolysosomes in podocytes after exposure to AGE-BSA. **a** Fluorescence microscopic analysis of podocytes transfected with plasmid constructs harboring LC3 fused with a tandem mRFP-GFP tag (tfLC3) and treated with Co-BSA, AGE-BSA (100 μg/ml), or rapamycin (RAP, 10 μM) for 12 h. **b** Quantitative data for green or red puncta per cell. **c** Quantitative data for yellow puncta or free red puncta per cell. The yellow puncta, which showed GFP and mRFP fluorescence signals, indicated autophagosomes that were not fused with lysosomes (arrowheads). The free red puncta indicate autolysosomes (arrows) where the GFP signal was quenched under acidic conditions. Compared with the control podocytes, the AGE-BSA-treated podocytes showed much more yellow puncta. However, podocytes exposed to RAP displayed increased autolysosomes. Scale bar, 10 μm. *p < 0.05 (for green or red puncta); ^#^*p* < 0.05 (for free red puncta)

Finally, we used double-tagged LC3 proteins (tfLC3) to monitor autophagic flux. This method relies on the different sensitivities of GFP and mRFP to acidic environments in the lysosome lumen to distinguish autophagosomes from autolysosomes^25,26^. Yellow puncta representing the colocalization of GFP (green) and mRFP (red) signals represents an autophagosome that is not yet fused with a lysosome. In contrast, a red signal was exclusively observed in autolysosomes, where the GFP signal was quenched because of the acidic environment. As shown in Fig. 7, after exposure to rapamycin, an autophagy inducer, the amount of free red puncta was enhanced, accompanying the increased in the number of free green puncta, thereby suggesting that the autophagic flux was unobstructed. However, the amount of yellow puncta was obviously increased, while few mRFP-positive free puncta could be observed in AGE-BSA-treated cells, indicating impaired degradation of autophagosomes.

### AGEs induced the impaired lysosomal degradation of podocytes

**Fig. 8:**
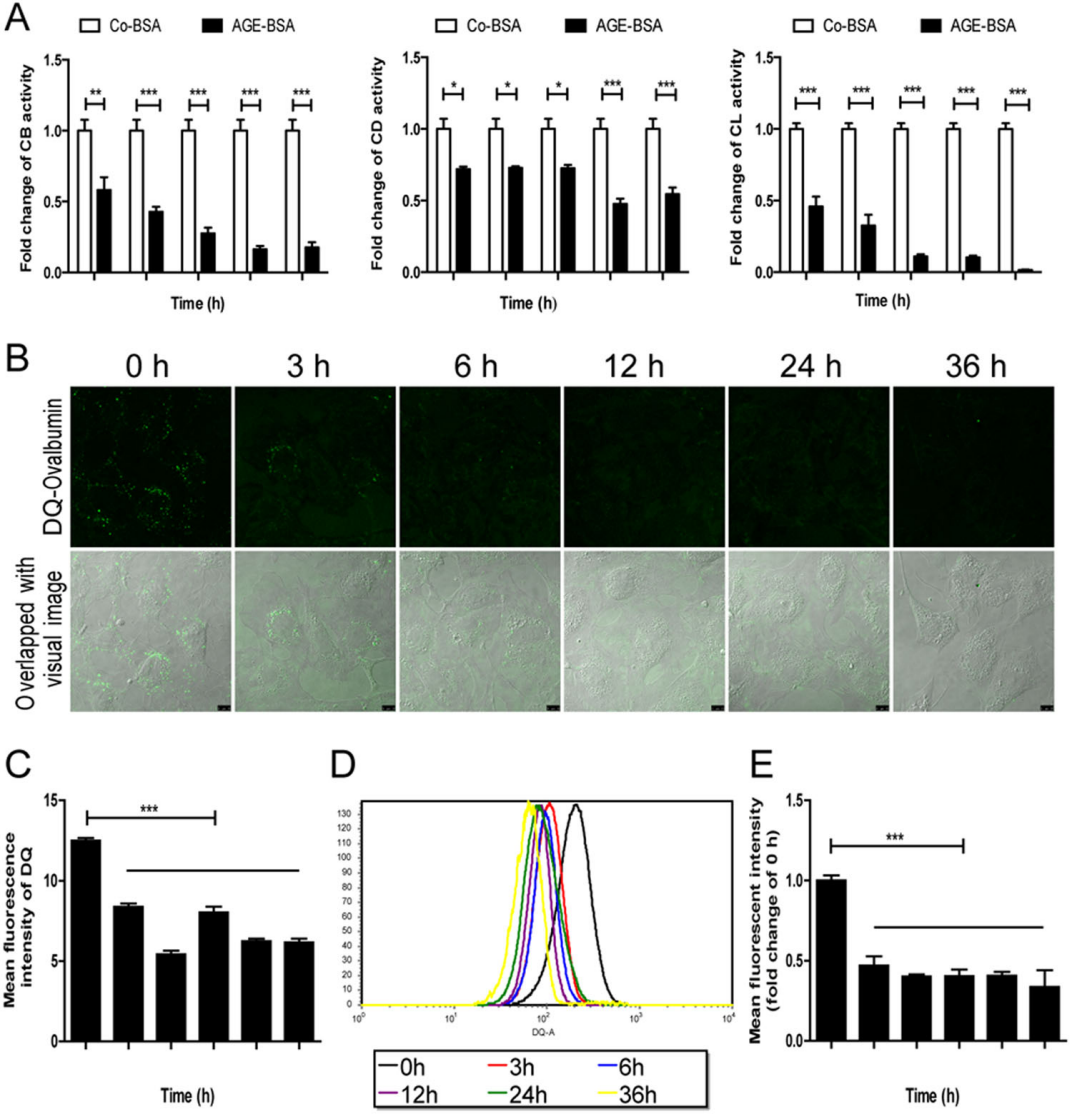
Effects of AGE-BSA on enzymatic activity and lysosomal degradation of DQ-ovalbumin in podocytes. **a** Markedly decreased proteolytic activity of cathepsin B, cathepsin D, and cathepsin L in podocytes after exposure to AGE-BSA for different times. **b**, **c** Cleaved fluorescent DQ-ovalbumin (green) obviously dropped in podocytes after exposure to AGE-BSA for 0, 3, 6, 12, 24, or 36 h. **d**, **e** The mean fluorescence intensity of the DQ-ovalbumin signal was significantly decreased after exposure to AGE-BSA for different times, as analyzed by flow cytometry. Scale bar, 10 μm; *p < 0.05; **p < 0.01; ***p < 0.001

The lysosomal-mediated degradation system is the key step of autophagy degradation. Therefore, we then examined the activities of lysosomal proteolytic enzymes. Compared with control-BSA (Co-BSA), a remarkable suppression in cathepsin B (CB), cathepsin D (CD), and cathepsin L (CL) activity was observed in the AGE-BSA group at 3, 6, 12, 24, and 36 h (Fig. 8a).

Furthermore, we used DQ-ovalbumin, a self-quenched substrate for proteases, to investigate the digestive function of lysosomes. DQ-ovalbumin requires enzymatic cleavage in acidic lysosome to generate a fluorescent product that can be monitored by fluorescence microscopy and flow cytometry^27^. The result showed that there was a significant drop in the DQ-ovalbumin puncta per cell after AGE-BSA exposure at all time points compared with that at 0 h (Fig. 8b, c), as assessed by fluorescence. Equally, the mean fluorescence intensity of DQ-ovalbumin in AGE-BSA-treated cells was lower than 0 h, as measured by flow cytometry (Fig. 8d, e). There were no significant differences among all time points after Co-BSA treatment. These results suggest that podocyte lysosome degradative potential is reduced when exposure to AGEs.

### AGEs triggered lysosomal membrane permeabilization in podocytes

**Fig. 9:**
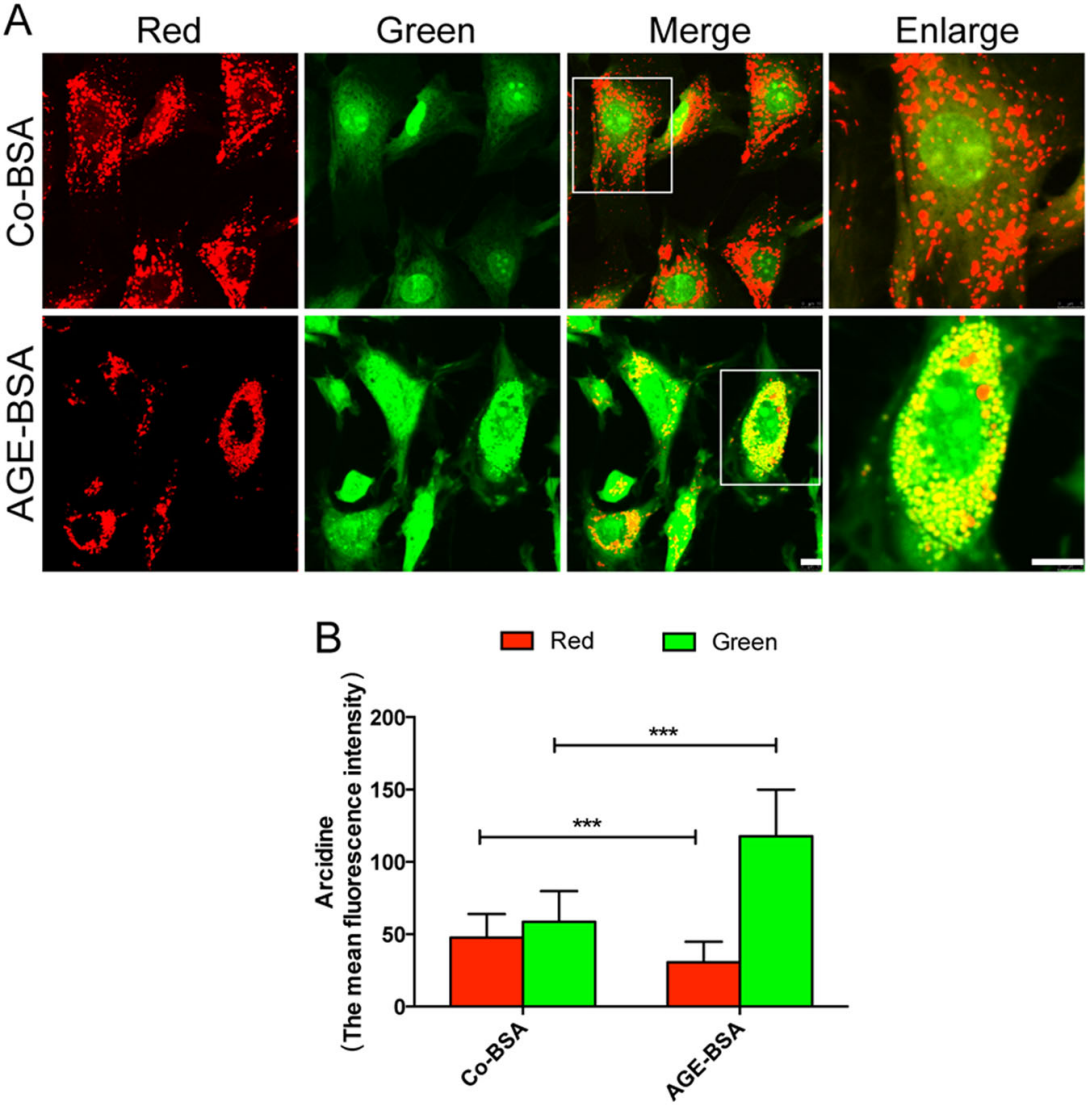
Effect of AGE-BSA on acridine orange distribution. **a** After exposure to 100 μg/ml AGE-BSA, podocytes were stained with acridine orange and viewed under a microscope. Concomitant with the increase of green fluorescence, a conspicuous reduction of red fluorescence was observed in podocytes treated with AGE-BSA. **b** Quantitative data for the mean fluorescence intensity of red or green signal. Scale bar, 10 μm. ***p < 0.001

Previous studies have shown that lysosomal membrane permeabilization (LMP) is a main cause of lysosomal dysfunction in renal tubular epithelial cells when exposed to AGE-BSA or urinary proteins^28,29^. Accordingly, we tested LMP by acridine orange (AO) redistribution. AO is a kind of lysomotropic metachromatic fluorescent dye that emits red fluorescence within lysosomes and green fluorescence in the cytosol. As shown in Fig. 9, numerous red granules were observed in the Co-BSA group, indicating AO located in the lysosomes and that the lysosomal membrane is integrated. However, concomitant with the increase of green fluorescence, a conspicuous reduction of red fluorescence was observed after the cells were treated with AGE-BSA, suggesting AO was released from lysosomes and redistributed in the cytoplasm. This indicated that AGEs triggered impaired lysosomal membrane integrity and LMP, since AO that is released from the lysosomes into the cytosol emits an enhanced green fluorescence in the cytoplasm.

**Fig. 10:**
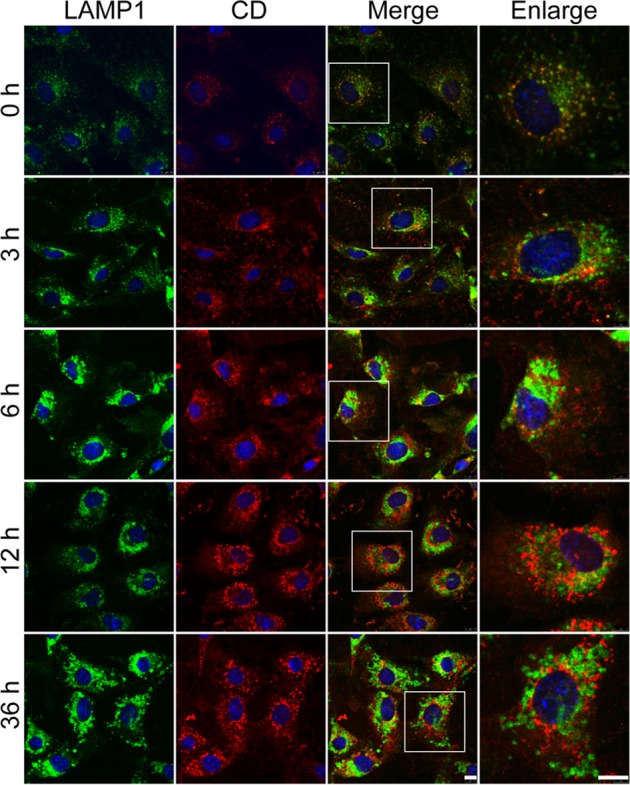
Changes in the distribution of cathepsin D (CD) and LAMP1 in podocytes after exposure to AGE-BSA. Immunofluorescence staining of LAMP1 and cathepsin D (CD) in podocytes after exposure to 100 μg/ml AGE-BSA for 0, 3, 6, 12, or 36 h. The red immunofluorescence showed the leakage of CD from lysosomes into the cytoplasm that was accompanied by dispersed LAMP1 (green fluorescence). Scale bar, 10 μm

Moreover, the permeabilization of the lysosomal membrane might result in leakage of cathepsins from lysosomes and diffusion into the cytoplasm^30^. Therefore, we then double-stained for lysosomal-associated membrane protein1 (LAMP1) and CD. LAMP1 is a lysosomal marker, and is primarily located at the lysosomal membrane. We found that CD granules were regularly distributed around the perinuclear, and most of them co-localized with LAMP1 in the Co-BSA group. However, in AGE-BSA-treated podocytes, the CD granules displayed an irregular and dispersed pattern. In the AGE-treated group, we observed irregular and larger cytoplasmic LAMP1 granules accompanied by a diffusion of CD (Fig. 10), indicating that lysosome content was leaked into the cytosol. Collectively, these results suggested that AGEs could induce podocyte LMP, which may contribute to lysosomal dysfunction.

### Lysosomotropic agent induced autophagic inhibition and podocyte injury

**Fig. 11:**
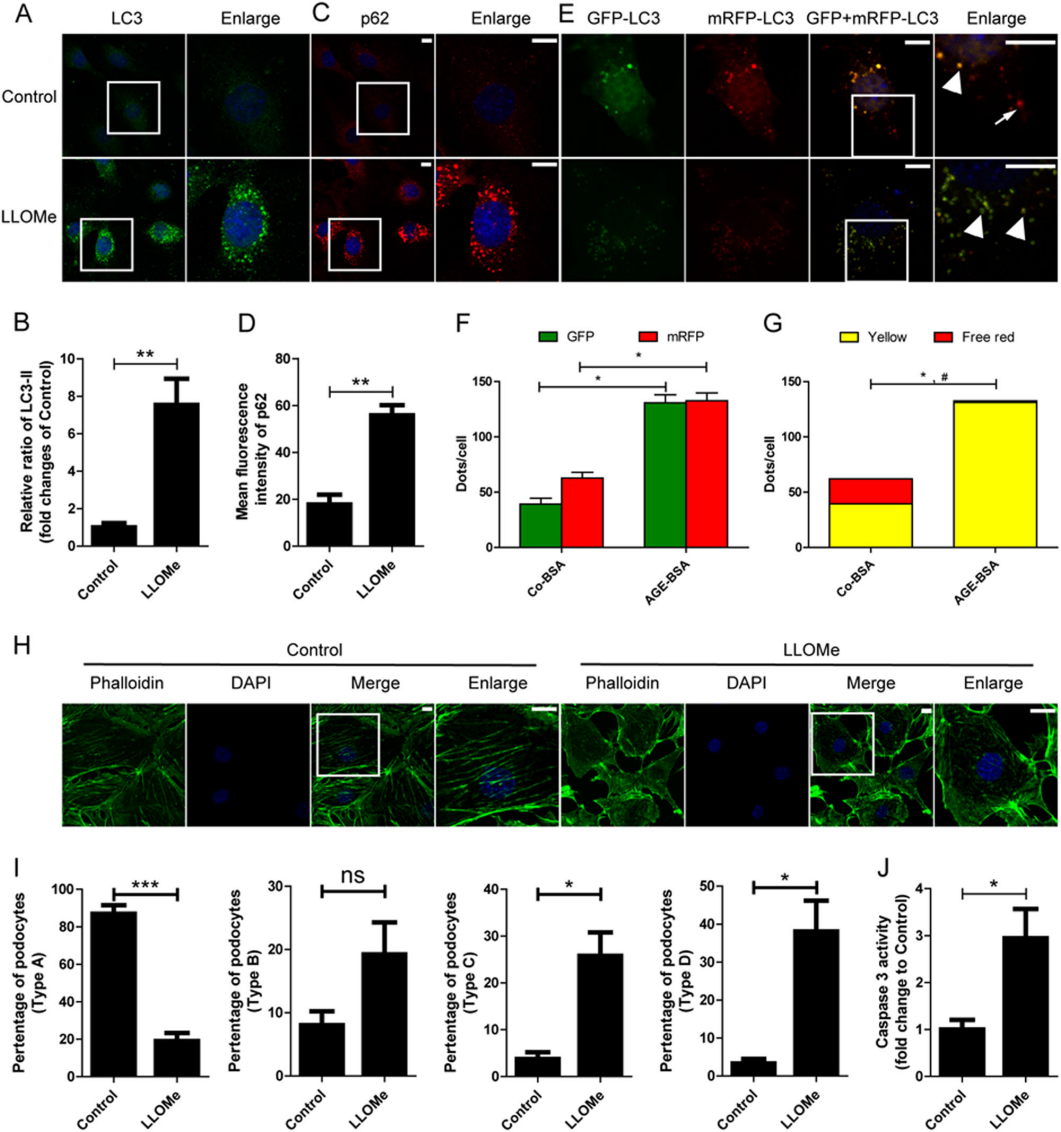
Effects of LLOMe on autophagy and cellular injury after exposure of podocytes to AGE-BSA. **a**, **c** Immunofluorescence staining of LC3 or p62 in podocytes after exposure to LLOMe. **b**, **d** Quantification of LC3 or p62 expression after exposure of podocytes to LLOMe. LLOMe induced an accumulation of LC3 or p62 in podocytes. **e** Fluorescence microscopic analysis of podocytes transfected with tfLC3 and treated with LLOMe. **f** Quantitative data for green or red puncta per cell. **g** Quantitative data for yellow puncta or free red puncta per cell. Autophagosomes (arrowheads), but not autolysosomes (arrows) were increased after exposure to LLOMe. **h** Immunofluorescence of F-actin cytoskeleton (green) showed loss of stress fibers and cortical actin staining after exposure of podocytes to LLOMe. **i** Quantification of the phalloidin staining. **j** The activity of caspase-3 was increased after exposure of podocytes to LLOMe. Scale bar, 10 μm

The autophagic activity and podocyte injury was then studied after exposure to l-leucyl-l-leucine-*O*-methyl ester (LLOMe), which is well known as the LMP inducer^31^. We found that LLOMe led to the increased expression of LC3-II and p62 when compared with controls (Fig. 11a–d), suggesting autophagic inhibition. After the cells were transfected with tfLC3, we found that the amount of yellow puncta was markedly increased, while few mRFP-positive free puncta could be observed in LLOMe-treated cells (Fig. 11e–g), indicating obstructed autophagic flux and imparied degradation of autophagosomes. As reorganization of the cytoskeleton is a characteristic of podocyte injury, we subsequently assessed the morphological changes of the F-actin cytoskeleton of podocytes by staining with fluorophore-conjugated phalloidin and quantifying the formation of stress fiber. Parallel bundles of stress fibers were observed in the actin distribution of control podocytes. The results showed that LLOMe induced polygonal cellular shape associated with a reduction in actin stress fibers, accompanied by the formation of cortical F-actin rings in the cytoplasm (Fig. 11h; quantified in Fig. 11i). Furthermore, apoptosis was increased in LLOMe-treated podocytes, characterized by an enhancement of caspase-3 activity (Fig. 11j). In general, the impact induced by LLOMe is in line with that induced by AGE-BSA, indicating that LMP may be a key node to trigger autophagic inhibition and cellular injury after exposure of podocytes to AGEs.

### Lysosomal function recovery activated autophagy and protected podocytes from AGEs-induced injury

**Fig. 12:**
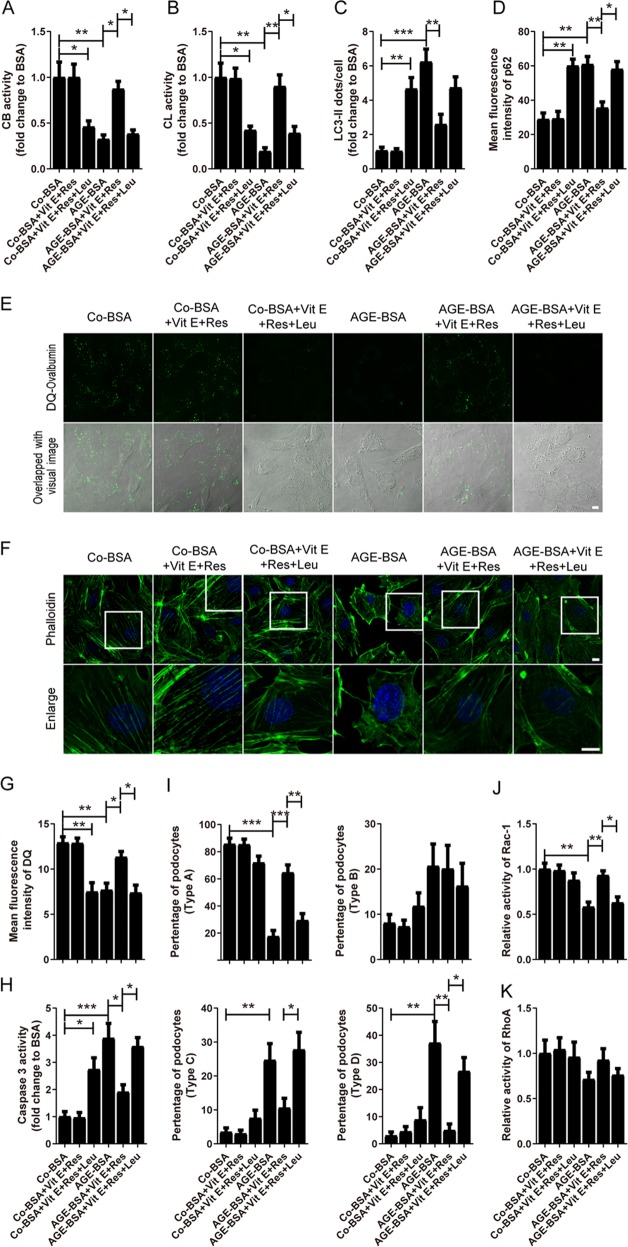
Effects of resveratrol plus vitamin E and leupeptin on autophagic-lysosomal pathway and cellular injury after exposure of podocytes to AGE-BSA. **a**, **b** The proteolytic activities of cathepsin B and cathepsin L were increased by resveratrol plus vitamin E treatment, which were blocked by leupeptin, in podocytes after exposure to AGE-BSA. **c**, **d** The accumulation of LC3-II or p62 was decreased by resveratrol plus vitamin E treatment, which was blocked by leupeptin, in podocytes after exposure to AGE-BSA. **e**, **g** The mean fluorescence intensity of the DQ-ovalbumin signal was enhanced by resveratrol plus vitamin E treatment, which was blocked by leupeptin, in podocytes after exposure to AGE-BSA. **f** Immunofluorescence of F-actin cytoskeleton (green) showed that the disrupted actin cytoskeleton was normalized by resveratrol plus vitamin E treatment, which was blocked by leupeptin, in podocytes after exposure to AGE-BSA. **h** The activity of caspase-3 was deceased due to the resveratrol plus vitamin E treatment, which was blocked by leupeptin, in podocytes after exposure to AGE-BSA. **i** Quantification of the phalloidin staining. **j**, **k** Rac-1 activity, but not RhoA activity, was decreased in AGE-BSA-treated cells, which could be rescued by resveratrol plus vitamin E treatment. Scale bar, 10 μm. *p < 0.05, **p < 0.01, ***p < 0.001

We next studied whether the AGE-induced autophagy-lysosomal pathway inhibition contributed to podocyte lesions. We found that resveratrol plus vitamin E treatment could increase the enzymatic activity of CB/CL and DQ-ovalbumin degradation in AGE-BSA-treated podocytes (Fig. 12a, b, e, g), suggesting an improvement of lysosomal function. Also, the accumulation of autophagic vacuole and autophagic substrate were decreased after treatment (Fig. 12c, d), indicating the recovery of autophagic activity. Notably, we found a striking reduction in actin stress fiber formation, with an accentuated cortical localization in AGE-BSA-treated cells, which was rescued by resveratrol plus vitamin E treatment (Fig. 12f; quantified in Fig. 12i). Furthermore, the increased podocyte apoptosis in AGE-BSA group, characterized by an enhancement of caspase 3 activity, was attenuated by resveratrol plus vitamin E treatment (Fig. 12h). It is well known that the actin cytoskeletal dynamics is mainly regulated by Rho family small GTPases, so the Rac-1 and RhoA activity was subsequently measured. We found that the Rac-1 activity, but not RhoA activity, was decreased in AGE-BSA-treated cells (Fig. 12j, k), indicating that AGE-BSA-induced actin-cytoskeletal disorganization was Rac-1-dependent. Interestingly, besides the recovery of lysosomal function and autophagic activity, resveratrol plus vitamin E treatment could rescue actin cytoskeleton changes by upregulating Rac-1 activity. However, all the cytoprotective effects were blocked after the lysosomal inhibitor leupeptin was added to disrupt the autophagic-lysosomal pathway, suggesting that LMP-induced autophagic inactivity is one of the main causes of podocyte injury during the progression of DKD.

## Discussion

In the present study, we demonstrated that the autophagy pathway was insufficient in podocytes under diabetic conditions. Moreover, AGE-induced LMP may be the main cause of impaired podocyte autophagy in DKD.

Autophagy is important for the survival of cells under stress. Recent studies have provided indirect and direct evidence demonstrating that autophagy was involved the pathogenesis of DKD, including tubulointerstitial lesions and glomerulosclerosis^2,32,33^. However, the type of renal intrinsic cells involved in the alteration of autophagy as well as the alterations that occur in the autophagic pathway must be further investigated for autophagy-based intervention strategies in the future. Some studies on mesangial cells not only showed that high glucose induced the accumulation of p62, but also showed a reduction in autophagy-related gene expression of patients with DKD, suggesting that autophagic activity was inhibited in mesangial cells in DKD^34,35^. In addition, our previous study showed that the autophagic activity was severely inhibited, and increased expression of LC3 and the accumulation of p62 were found in the renal tubular epithelial cells in DKD^28^. It is well known that podocytes exhibit high levels of basal autophagy under physiological conditions, and that dysfunction of autophagy was associated with podopathy, especially typical podopathy DKD^36,37^. Previous studies have shown that high glucose could inhibit the activity of podocyte autophagy by downregulating the expression of LC3-II and upregulating the expression of p62^38^. Moreover, high glucose also suppresses the expressions of Beclin-1 and Atg5-12 in podocytes^33,39,40^. However, autophagic process is very complicated, while most of the studies mainly focus on the upstream of autophagic pathway. Although previous study showed that damaged lysosomes occurred in podocytes of DKD, a blockage of autophagy induction induced by some unknown etiological factor was reported as the main cause^16^. In the present study, we demonstrated predominately the downstream alteration in autophagic pathway induced by AGEs. Through in vivo and in vitro experiments, we showed that autophagic vacuole and autophagic substrate accumulated in podocytes after treatment with AGEs. However, AGEs did not lead to autophagy induction since either Beclin 1 or ATG7 mRNA and protein levels were not changed, as well as LC3B mRNA was not upregulated. These results supported the hypothesis that the accumulation of autophagic vacuoles was mainly due to the blockage of a pathway downstream of autophagy.

The node in the downstream of autophagy pathway that leads to an inhibition of autophagic activity in renal intrinsic cells in DKD has not been well studied. Our previous studies have shown that after exposure to urinary protein or AGEs, LMP was triggered and resulted in lysosomal dysfunction, which may be the key node in the impaired autophagy in renal tubular cells^28,29^. We next investigated whether lysosomal impairment involved the same key node as the inhibition of autophagy in podocytes. We used tfLC3 to monitor autophagic flux and found that most autophagosomes were not degraded after AGE treatment. Next, we observed a significant decrease of lysosomal enzyme activities and degradative potential of DQ-ovalbumin in AGE-treated podocytes. To assess the cause of the decreased lysosomal degradation, we performed AO staining and found that, accompanied by green fluorescence enhancement, the red fluorescence decreased significantly after exposure to AGE-BSA, suggesting the destruction of lysosomal membrane integrity. In addition, a leakage of enzymes and an irregular arrangement of LAMP1 were observed in AGE-treated podocytes. These results indicated that LMP occurred in podocytes under DKD condition. To verify whether autophagic inhibition was triggered by LMP after exposure to AGEs, the LMP inducer LLOMe was utilized^31,41^. It was shown that the lysosomotropic agent resulted in an accumulation of autophagic vacuoles and substrates, an increase in autophagosomes but not autolysosomes, as well as podocyte injury, which simulated the action of AGEs. Interestingly, the treatment of resveratrol plus vitamin E, which are typical antioxidants, largely reversed the AGE-induced autophagic inhibition and cellular injury by increasing lysosomal function, However, the protective effects of resveratrol plus vitamin E treatment was blocked by lysosomal inhibitor leupeptin. Thus, we propose that LMP results in lysosomal dysfunction, which is a key node resulting in insufficient autophagy of podocytes and may be a common etiologic factor in the failure of renal intrinsic cells in DKD.

However, the mechanism by which DKD induces LMP in podocytes remains unclear. It has been reported that LMP was associated with oxidative stress and ROS overload^42^. And our recent studies have suggested that urinary protein and AGEs might partly trigger LMP by mediating oxidative stress in renal tubular epithelial cells^28,29^. In the present study, two typical antioxidants were used in combination, and the protective effects were achieved on podocytes exposed to AGEs. And previous studies have reported that both resveratrol and vitamin E have the effect of improving oxidative status under diabetic conditions^43,44^. Therefore, we assume that AGE-induced oxidative stress contributes to the LMP of podocytes. Furthermore, the pathophysiology of DKD is closely associated with the disorder of various other signaling pathways, inluding inflammation, energy and nutrient-sensing pathways (AMP-activated protein kinase, the mammalian target of rapamycin, and sirtuin 1), etc.^45–47^. Some of them might play a role in lysosomal membrane instability although it remains uncertain. Thus the exact reason and underlying mechanisms for the induction of LMP in DKD need further studies^48^.

In conclusion, LMP induces lysosomal dysfunction, which is a key node of insufficient autophagy of podocytes in DKD, and is probably a common phenomenon in renal intrinsic cells in DKD. Therefore, our study may contribute to the development of lysosome-based intervention strategies for DKD.

## Materials and methods

### Patients

The Institutional Review Board of the Affiliated Hospital of Guangdong Medical University approved this study. Kidney tissue specimens were obtained from biopsy-proven diabetic nephropathy patients (*n* = 11), while the kidney specimens obtained from patients with mild urinary protein excretion or only hematuria and characterized with a minimal change in histology (*n* = 5) were used as controls.

### Cell culture and treatments

Conditionally immortalized mouse podocytes were originally provided by Dr. Jochen Reiser from Rush University Medical Center and cultured in DMEM medium (Gibco, New York, USA) supplemented with 10% fetal bovine serum (Gibco, New York, NY, USA) and 100 U/ml penicillin–streptomycin (Gibco) in the presence of mouse recombinant γ-interferon (γ-INF, Millipore, Billerica, USA) at 33 °C in 5% CO_2_ (permissive conditions). To induce differentiation, podocytes were maintained at 37 °C without IFN-γ (non-permissive conditions) for 10–14 days. The cells were exposed to 50, 100, and 200 μg/ml nonglycated Co-BSA or AGE-BSA (Millipore Sigma, St Louis, MO, USA) for 12 h. Next, we chose the concentration of 100 μg/ml to treat cells for 0, 3, 6, 12, 24, and 36 h. LLOMe (Santa Cruz Biotechnology, Santa Cruz, CA) was included as a positive control for LMP (1 mM for 1 h). The protein levels of LC3B, Beclin 1 and ATG7, LAMP1, CD, p62 (Abcam, Cambridge, MA, USA), as well as the activities of CB, CD, and CL were then measured. Additionally, the mRNA levels of LC3B, Beclin 1, and ATG7 were examined. Moreover, ovalbumin dequenching and AO uptake and redistribution were tested. Subsequently, after exposure to Co-BSA and AGE-BSA, the cells were incubated with 100 nM bafilomycin A1 (Abcam, Cambridge, MA, USA), 200 μg/ml leupeptin (Sigma-Aldrich, St. Louis, MO), or 10 μM chloroquine (Sigma, St. Louis, MO) for LC3-II or p62 turnover assay at the 12 h time point. After treatment with resveratrol plus vitamin E for 24 h, autophagic-lysosomal pathway and cell injury were tested. Vitamin E (DL-α-tocopherol; Sigma-Aldrich, Poole, UK) was originally dissolved in 100% EtOH and further diluted in growth medium to result in a final concentration of 50 μM. Resveratrol (Sigma-Aldrich, St. Louis, MO) was used at 100 μM. The activity of caspase 3 was determined by colorimetric caspase 3 assay kit (Abcam, Cambridge, MA) according to the manufacturer’s protocol.

### Enzymatic assay

Fluorescence-based assay kits (BioVision, San Francisco Bay, USA) were used to measure the activity of CB, CD, or CL. After cleavage of the synthetic substrate by the cell lysate, the released fluorescence was quantified using a fluorescence plate reader according to the manufacturer’s instructions.

### Rac-1 or RhoA activation assay

In podocyte lysates, active Rac-1 or RhoA was measured by G-LISA Rac-1 or RhoA Activation Assay Biochem kit (colorimetric assay, Cytoskeleton, Denver, CO) following the manufacturer’s instruction. The signal was measured at 490 nm with a microplate reader. Results were expressed as fold changes to Co-BSA.

### Immunofluorescence study

Paraffin-embedded sections of patient kidney specimens were deparaffinized, rehydrated, subjected to antigen retrieval with 10 mmol/L sodium citrate buffer at pH 6.0 and heat, permeabilized with 0.25% PBS–Tween, and blocked with 3% bovine serum albumin. Podocytes were grown on glass coverslips in 12-well plates and cultured as described previously. The coverslips were washed briefly in PBS, fixed with 4% paraformaldehyde, and permeabilized with 0.5% Triton X-100 prior to blocking in 5% BSA, and then incubated overnight at 4 °C with a primary rabbit anti-LC3B, rabbit anti-LAMP1, rabbit anti-Beclin 1, mouse anti-CD (Abcam, Cambridge, MA, USA), rabbit anti-ATG7 (MBL, Nagoya, Japan), and mouse anti-p62 (Santa Cruz Biotechnology). On the following day, Alexa Fluor-488 donkey anti-rabbit and Alexa Fluor 594 donkey anti-mouse IgG antibodies (Invitrogen) were used for staining. To stain the cytoskeleton, podocytes were incubated with anti-FITC-phalloidin (Cytoskeleton, Denver, CO, USA) at room temperature in the dark for 30 min. DAPI was used to stain the nuclei. The images were taken with a TCS SP5 II confocal microscope (Leica Microsystems, Wetzlar, Germany). Expression levels of LC3-II and p62 in 15–20 glomeruli for each human were first graded on a scale of 0–4, and the average of the scores was subsequently calculated. The puncta/cell and average fluorescence intensity values for LC3-II and p62 were calculated from at least 50 cultured cells for each group blindly by two independent investigators, and the slides were mixed with a set of normal slides to ensure that the examiner was blinded to its information. In order to quantify the formation of stress fiber in podocytes, four major types were grouped after stained with phalloidin, as described previously^49^. In brief, type A: more than 90% of cell area filled with thick cables; type B: at least two thick cables running under nucleus and rest of cell area filled with fine cables; type C: no thick cables, but some fine cables present; type D: no cables visible in the central area of the cell.

### Transmission electron microscopy

After exposure of podocytes to 100 μg/ml for 12 h, the cells were collected and examined by Electron microscopy as described previously^50^. In short, specimens were fixed in 2.5% glutaraldehyde in 0.1 M sodium phosphate buffer, and then dehydrated in a graded ethanol series and embedded. After cut and stained, the ultrathin sections were examined using a JEM-1400 electron microscope (JEOL, Tokyo, Japan).

### Real-time quantitative PCR

Total RNA was extracted by RNX-Plus solution (Takara Bio INC, Japan) according to the manufacturer’s instructions. The cDNA was synthesized at a final volume of 20 μl using cDNA synthesis kit (Takara Bio INC, Japan). Quantitative real-time polymerase chain reaction (qRT-PCR) analyses were performed using a SYBR Green mix in the Real-Time PCR System (Takara). Relative gene expression data were calculated using the comparative threshold cycle method (ΔΔCt) with ACTB as housekeeping genes. To assess the specificity of each amplification, dissociation analysis was performed in every run. The primer sequences were as follows:

*LC3B*: forward, 5′-GGATATAGGTCACCCCTCAG-3′,

reverse, 5′-GTTAAAGGAGTTCCTGTCACC-3′;

*Beclin-1*: forward, 5′-CTGAAACTGGACACGAGCTTCAAG-3′,

reverse, 5′-TGTGGTAAGTAATGGAGCTGTGAGTT-3′;

*ATG7*: forward, 5′-ATGCCAGGACACCCTGTGAACTTC-3′,

reverse,5′-ACATCATTGCAGAAGTAGCAGCCA-3′.

### Plasmid transfection

Podocytes were transfected with the mRFP-GFP tandem fluorescent-tagged LC3 (tfLC3) plasmid (Addgene, Cambridge, MA, USA) using Lipofectamine 3000 Transfection Kit (Invitrogen) according to the manufacturer’s instructions. After transfection, the cells were treated with Co-BSA and AGE-BSA to assess autophagosome and autolysosome formation as described previously^50^.

### AO staining

After the designated treatments, the cells were incubated with 2 mg/ml AO (Sigma, St Louis, MO, USA) for 15 min at 37 °C. The fluorescence was examined using a confocal microscope with the excitation wavelength set at 488 nm and emission of 530 and 650 nm.

### Ovalbumin dequenching assay

After exposure to AGE-BSA for 12 h, podocytes were incubated with 10 μg/ml DQ-ovalbumin (Invitrogen) for an additional 2 h at 37 °C. Then the cells were washed with PBS and fixed with 4% paraformaldehyde. The mean fluorescence intensity of greenfluorescent DQ-ovalbumin puncta in individual podocytes was calculated and presented in the figures, and a FACS analysis was performed simultaneously.

### Western blot analysis

Western blot analysis was performed as described previously^28^. Primary antibodies against LC3B (Sigma), p62 (Santa Cruz), β-Actin and tubulin (Abcam), as well as appropriate HRP-conjugated secondary antibodies (Beyotime Institute of Biotechnology) were used.

### Statistical analysis

All experiments were performed at least three times and statistical tests were performed using SPSS 16.0. All data were expressed as the means ± standard error of the mean (S.E.M.), counts, or percentage. Two-group comparisons were performed using an independent samplest test unless otherwise indicated. Multiple group comparisons were performed using analysis of variance followed by Bonferroni or Dunnett’s post-hoc tests. Differences with a *p* value < 0.05 were considered statistically significant.
